# Autophagy: Mechanisms and Therapeutic Potential of Flavonoids in Cancer

**DOI:** 10.3390/biom11020135

**Published:** 2021-01-21

**Authors:** Xuening Pang, Xiaoyi Zhang, Yuhuan Jiang, Quanzhong Su, Qun Li, Zichao Li

**Affiliations:** 1 Institute of Biomedical Engineering, College of Life Sciences, Qingdao University, Qingdao 266071, China; 2018025216@qdu.edu.cn (X.P.); 2018025210@qdu.edu.cn (Y.J.); 2 Department of Pharmacology, School of Pharmacy, Qingdao University, Qingdao 266021, China; 13061477181@163.com (X.Z.); quanzhongsu0930@163.com (Q.S.); 3 College of Chemistry and Chemical Engineering, Qingdao University, Qingdao 266071, China; qunli@qdu.edu.cn; 4 Qingdao Balanson Biotech Co., Ltd., Qingdao 266071, China

**Keywords:** flavonoids, autophagy, anti-cancer effects, apoptosis, chemoresistance, mechanism

## Abstract

Autophagy, which is a conserved biological process and essential mechanism in maintaining homeostasis and metabolic balance, enables cells to degrade cytoplasmic constituents through lysosomes, recycle nutrients, and survive during starvation. Autophagy exerts an anticarcinogenic role in normal cells and inhibits the malignant transformation of cells. On the other hand, aberrations in autophagy are involved in gene derangements, cell metabolism, the process of tumor immune surveillance, invasion and metastasis, and tumor drug-resistance. Therefore, autophagy-targeted drugs may function as anti-tumor agents. Accumulating evidence suggests that flavonoids have anticarcinogenic properties, including those relating to cellular proliferation inhibition, the induction of apoptosis, autophagy, necrosis, cell cycle arrest, senescence, the impairment of cell migration, invasion, tumor angiogenesis, and the reduction of multidrug resistance in tumor cells. Flavonoids, which are a group of natural polyphenolic compounds characterized by multiple targets that participate in multiple pathways, have been widely studied in different models for autophagy modulation. However, flavonoid-induced autophagy commonly interacts with other mechanisms, comprehensively influencing the anticancer effect. Accordingly, targeted autophagy may become the core mechanism of flavonoids in the treatment of tumors. This paper reviews the flavonoid-induced autophagy of tumor cells and their interaction with other mechanisms, so as to provide a comprehensive and in-depth account on how flavonoids exert tumor-suppressive effects through autophagy.

## 1. Introduction

### 1.1. Flavonoids

Flavonoids, as naturally occurring polyphenols, are widely present in a variety of plants employed for both medicine and food [1]. In ancient times, they were considered of great medicinal value and established a vital therapeutic effect in the medicinal system [2]. Flavonoids exhibit potent activities in the chemoprevention and treatment of many chronic human diseases, such as tumors, possessing an inestimable development potential.

Emerging evidence suggests that the anticarcinogenic mechanisms of flavonoids include proliferation inhibition, the induction of apoptosis, autophagy, necrosis, cell cycle arrest, senescence, the impairment of cell migration, invasion, tumor angiogenesis, and the reduction of multidrug resistance in tumor cells [2,3,4,5]. In addition, flavonoids have a cytoprotective or cytotoxic effect on tumor cells through acting with multiple targets participating in multiple pathways associated with the development of cancer. For example, protective autophagy induced by flavonoids resists its anti-tumor effects, while autophagic cell death induced by flavonoids exerts a synergistic anti-tumor effect. Flavonoids serve as chemo-preventive and therapeutic agents for diverse cancers through manipulating autophagy. For instance, Apigenin has a preventive effect on UV-induced skin cancer by apigenin-induced autophagy through stimulating AMP-activated protein kinase (AMPK) or inhibiting protein kinase B (AKT) activation in keratinocytes (both human and mouse keratinocyte cell lines and primary normal human epidermal keratinocytes) to inhibit UV-mediated mammalian target of rapamycin (mTOR) activation [6,7].

**Figure 1:**
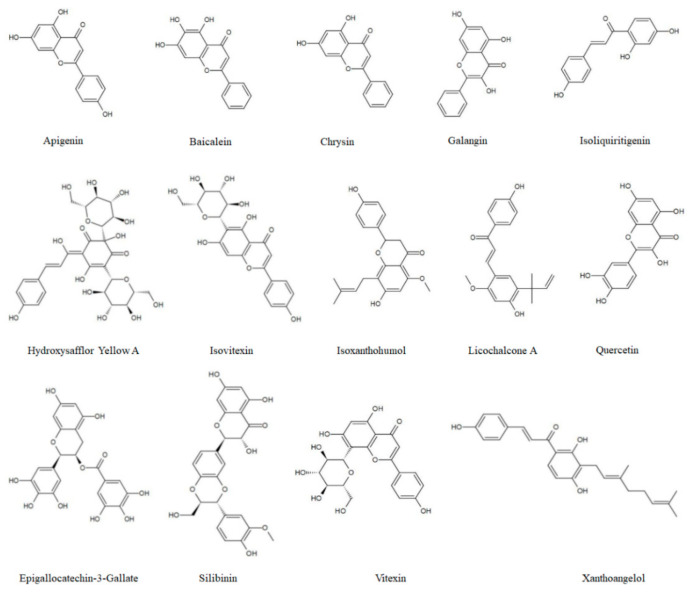
The chemical structure of several flavonoids that target autophagy.

Autophagy, which is the pathway of cell death induced by flavonoids, is acknowledged to be an effective strategy for the development and improvement of anticancer drugs [3,8]. Autophagic signals commonly form crosstalk with other mechanisms, comprehensively creating a complex network. Accordingly, this paper reviews the comprehensive understanding of how flavonoids display their co-chemotherapeutic effects by regulating autophagy. The chemical structures of several flavonoids introduced in this paper are shown in Figure 1.

### 1.2. Autophagy Mechanisms

Autophagy, which is a common life phenomenon and an important cell survival mechanism in eukaryotes, can maintain cell metabolism and homeostasis under stress by capturing and degrading its own redundant and damaged organelles, as well as cytoplasmic protein aggregates. It is mainly divided into three types: Macroautophagy; microautophagy; and chaperone-mediated autophagy. Macroautophagy is a metabolic process in which cells wrap proteins or organelles to form autophagososomes through bilayer membranes. Then, the outer membrane fuses with the lysosomal membrane to form autophagolysosomes, and finally degrades the wrapped contents by hydrolyzing enzymes. The other two types of autophagy are just different in their mode of delivering the particular cargo to be degraded by lysosomes [9], which will not be discussed in this article.

**Figure 2:**
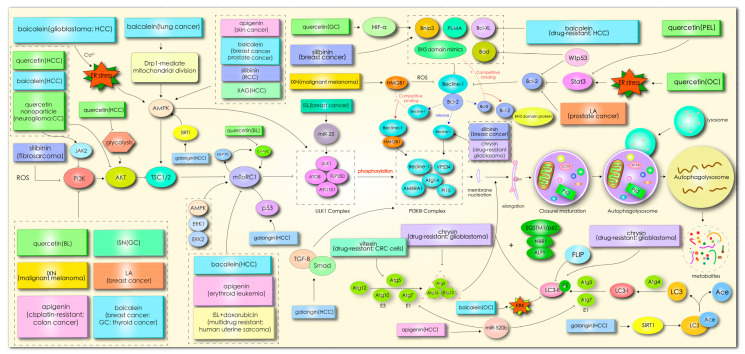
Mechanism through which flavonoids induce cancer cell autophagy. Many autophagic regulators may determine the process of autophagy. Phosphoinositide 3-kinase (PI3K)/protein kinase B (AKT) can stimulate the mammalian target of rapamycin (mTOR), which is negatively regulated by mitogen-activated protein kinase (MAPK), to suppress activation of the unc-51-like kinase (ULK)1/2 complex. Additionally, MAPK can be directly activated by the ULK1/2 complex in an independent mTOR manner. Subsequently, the ULK1/2 complex triggers membrane nucleation by phosphorylating the PI3K III complex, including phosphorylation beclin-1, activation AMBRA1, Atg14, and p115. The PI3K III complex recruits two interrelated ubiquitin-like protein (UBL) binding systems (the Atg12-Atg5-Atg16 system and LC3 system) to regulate membrane elongation and autophagosome expansion. Damaged proteins and organelles are brought into the autophagosome through the LC3 complex containing p62/SQSTM1. If the damaged protein is not transported to the autophagosome, p62/SQSTM1 accumulates in the cell, exacerbating endoplasmic reticulum stress. mTOR, as an important target for autophagy, is targeted by several tumor regulators, such as TSC1/2, PTEN, JAK2, silent information regulator 2 homolog 1 (SIRT1), AMP-activated protein kinase (AMPK), p53, and reactive oxygen species (ROS). In particular, heat shock proteins (HSPs) can stabilize proto-oncogene c-Myc activation, upregulating mTOR expression and promoting cell survival. MicroRNAs (miRNAs), by targeting various target genes or proteins such as miR-25, directly target the ULK1 complex and miR-520b targets the Atg7 protein, inhibiting autophagy to improve the chemosensitivity. BH3-only proteins (Bad, Bcl-2 adenovirus E1B 19-kDa-interacting protein 3 (BNIP3), Puma, Bcl-XL, and BH3 domain mimics) bind to the anti-apoptotic protein Bcl-2, directly regulating autophagy and apoptosis. Similarly, the high-mobility group box 1 protein (HMGB1) competitively combines with Beclin-1 to form a complex promoting the autophagy process and chemotherapy resistance. Transforming growth factor (TGF)-β1 exerts antitumorigenic functions mediated by Smads, and increases beclin1 levels, which interacts with the increased levels of Bcl-XL and decreased levels of Bcl-2 to induce autophagy. SIRT1 regulates the deacetylation of autophagy-associated proteins (LC3 and forkhead box class O (FoxO)) to trigger autophagy. Additionally, some signal pathways and alteration of the metabolic environment can mediate the autophagy signals of tumor cells, including endoplasmic reticulum (ER) stress, death-associated protein kinase (DAPK)-related protein kinase (Drp1)-dependent mitochondrial fission, the ERK signal pathway, and glycolysis. The mechanism of flavonoids regulating autophagy is diverse in different tumor cells. Numerous flavonoids regulate the autophagy of tumor cells by targeting the mTOR signal pathway. Moreover, quercetin, silibinin (SIL), isoxanthohumol (IXN), chrysin, baicalein, Licochalcone A (LA), and galangin stimulate autophagy by targeting apoptosis-related proteins or HMGB1 to regulate the interaction between Bcl-2 and beclin-1. Vitexin, chrysin, baicalein, apigenin, and galangin interfere with UBL binding systems to influence membrane elongation and expansion. Collectively, flavonoids may control tumor progression by manipulating autophagy.

In mammals, more than 30 autophagy-related genes (ATGs) have been discovered, and they are mainly responsible for the formation of functional complexes which serve as the core pathway of autophagy [10]. When cells enter into a state of stress, silencing key ATG genes, such as ATG3, ATG4, Beclin1/ATG6, ATG10, and ATG12, can induce tumor transformation [11]. Homologs of Atg1 (unc-51-like kinase (ULK)1 or ULK2), ATG13, and scaffold protein FAK interacting protein 200 kD (FIP200, the homologous protein of yeast ATG17) form ULK complexes, receiving signals from nutritional conditions and recruiting downstream Atg proteins participating in autophagosome formation [11,12]. In the case of nutritional deficiency, the inactivation of rapamycin complex mTORC1 can induce activation of the unc-51-like kinase (ULK1/2) complex, which phosphorylates FIP200 in the process of autophagy initiation [13]. In addition, mTOR, as the main negative regulator of autophagy in cancer cells, responds to specific signals between cell growth and autophagy, such as the nutritional status, growth factors, stress, and so on [11]. Subsequently, the activated ULK complex phosphorylates the PI3K-Beclin1 protein complex (composed of Beclin1, ATG14L, p150, and Vps34), which induces nucleation and initial autophagosomal membrane formation [12,14]. Additionally, the complex recruits two interrelated ubiquitin-like protein (UBL) binding systems to regulate membrane elongation and autophagosome expansion [15]. Autophagy-associated proteins ATG5 and ATG12 interact with ATG16 to form AG12-ATG5-AG16 complexes with an E3-like enzyme function. Simultaneously, ATG4 hydrolyzes LC3 to produce the LC3-I precursor molecule, which binds to E1 ligase (ATG7) and transfers to E2 ligase (ATG3). Finally, the ATG12-ATG5-ATG16L1 complex promotes the coupling of LC3-I with phosphatidylethanolamine (PE), resulting in LC3-II remaining on mature autophagosomes to end the elongation reaction [14,16]. In addition, the LC3-II left on the autophagosome can recruit a selective substrate by binding to the autophagy receptor protein p62/SQSTM (P62). Once the two bind to the LC3-II protein, part of the protein is recycled into LC3-I under the action of Atg4B [17]. Acid lysosomes combine with autophagosomes to form autophagolysosomes and then degrade the contents. The specific mechanism is shown in Figure 2.

### 1.3. Role of Autophagy in Cancer

There is a complicated relationship between the changes associated with autophagy and therapy from the development of a tumor, depending on the cancer type [18]. Autophagy plays different roles in different cells, external factors of the same cell, or stages of tumor development [19]. The baseline level of autophagy in normal cells is relatively low, yet there are inconsistent levels of autophagy in various tumor cells [20]. Studies have shown that the autophagy level in certain tumor cells is lower than in normal cells, even in the case of hypoxia or nutritional deficiency, and the autophagy level cannot be enhanced due to monoallelic deletion of the BECN1 gene, such as in breast cancer, ovarian cancer, and prostate cancer cells; however, for most tumor cells or advanced cancer cells, a higher level of basic autophagy is needed to meet the higher metabolic demands [20,21,22].

In normal cells, the functions of autophagy include the removal of abnormally folded proteins and damaged organelles for balancing energy sources, the prevention of DNA damage, a reduction of the cellular stress response, and tumor incidence. However, in cancers, this function of autophagy plays the opposite role, which may facilitate the survival of tumor cells [20]. Tumor cells suffer from various metabolic pressures in the process of growth, from hypoxia to nutritional deficiency, and even chemotherapy intervention, while the existence of autophagy means that normal cells or tumor cells have metabolic plasticity, so that cells can adapt to a series of harsh environments [9,23]. For example, in the model of fibroblast-breast cancer cell MCF-7 co-culture for simulating the tumor microenvironment, autophagy induction could reduce the mitochondrial mass in fibroblasts, resulting in aerobic respiratory inactivation and glycolysis conversion to provide rich metabolites (lactic acid and pyruvate) supporting the oxidative phosphorylation of tumor cells [24]. Furthermore, autophagy functions as a tumor promoter through participation in diverse metabolic pathways to degrade various substrates and provide fuel for almost all aspects of carbon metabolism, such as the degradation of carbohydrates into sugars and DNA into nucleosides to promote glycolysis, and the conversion of proteins into amino acids and lipids into fatty acids to facilitate the tricarboxylic acid (TCA) cycle [9]. Apparently, autophagy suppression during tumor progression can destroy tumor metabolism, leading to adverse metabolic consequences, including impaired mitochondrial metabolism, cholesterol accumulation, redox imbalance, nucleotide pool depletion, and a reduced energy charge; in some cases, it may even lead to impaired tumor growth and tumor cell death [9].

Autophagy has an undesirably complicated role in promoting tumor initiation, growth, survival, deterioration, and metastasis. At the early stage of the tumor, autophagy restrains tumorigenesis and silencing autophagy-related genes will lead to DNA damage and genomic instability, triggering initial tumorigenesis [19]. In certain tumors, such as those caused by chronic tissue injury or inflammation, autophagy knockout increases tumor initiation in the pancreas and liver of mice. However, autophagy is still required for the tumor to develop to the malignant stage. Therefore, autophagy removal is not conducive to the growth of malignant tumors, but contributes to the formation of benign tumors [23].

## 2. Autophagic Cell Death

Autophagic cell death (ACD) is a novel approach of cell death differing from apoptosis and is called type II programmed cell death. It is mainly characterized by numerous autophagosomes and autophagy lysosomes in the cytoplasm, the elimination of a bunch of cytoplasm, and accompanying intact nuclei [25]. Chemical inhibitors (3-methyladenine (3-MA) or wortmannin) or genetic ablation (knockout or small interfering RNA (siRNA) silencing of autophagy-related genes) inhibiting autophagy does not block cell death; a process that should not be designated as autophagic cell death [26], but just as protective autophagy hindering starvation-induced necrosis, apoptosis, and cell death [27]. ACD is the consequence of excessive autophagy, independent of an effect on activity of the Caspase family [28]. When autophagy occurs in a certain context, the intensity and duration of autophagy exceed the threshold required for cell survival, which will cause ACD [29].

Autophagic cell death can be regulated by a variety of signals, such as phosphoinositide 3-kinase (PI3K)/AKT/mTOR, AMPK, mitogen-activated protein kinase (MAPK), Beclin-l signal, Wnt/β-catenin, and other signal pathways [30]. These signal pathways interfere with each other to comprehensively stimulate autophagy in tumor cells. mTOR is an evolutionarily conserved serine/threonine protein kinase, which regulates multiple cellular functions by phosphorylating downstream target proteins and it plays an important role in autophagy and apoptosis [31]. In addition, the forkhead box class O (FOXO) protein family includes highly conserved transcription factors activating the expression of autophagy-related genes, such as Gabarapl1 and Atg12, and suppressing the activity of mTORC1 [32]. The ultra-violet radiation resistance-associated gene protein (UVRAG) has been found to mediate autophagy by associating with Beclin-1 to suppress tumorigenicity [33]. P53, Bcl-2, death-associated protein kinase (DAPK), DAPK-related protein kinase (DRP-1), insulin-like growth factor-1 (IGF-1), calcium ions, and GTP enzymes are also involved in the regulation of autophagy in tumor cells [30,34,35]. Sufficient studies have found that flavonoids regulate the autophagy of tumor cells through different signal pathways or modify the molecular mechanism, such as PI3K/Akt/mTOR, AMPK, MAPK, Beclin-l, Wnt/β-catenin, etc., and induce excessive autophagy and death of cancer cells, as shown in Table 1.

As a cancer chemopreventive agent, apigenin is able to suppress tumor cell proliferation, movement, angiogenesis, the cell cycle, and multidrug resistance by inducing autophagy, so it plays a prominent role in cancer chemoprevention [36,99]. Zhang et al. reported that apigenin induced autophagic death in thyroid papillary carcinoma BCPAP cells, which stimulated the production of reactive oxygen species (ROS) and DNA damage, and downregulated cell division cycle 25c (Cdc25C) expression, resulting in G2/M phase arrest and autophagic death. The autophagy inhibitor 3-MA saved cells from apigenin-induced cell death [37]. Baicalein, which is a flavonoid component rich in the root of baicalin, blocks thyroid cancer cell proliferation in a dose-dependent manner and induces autophagic cell death by regulation of the nuclear factor kappa B (NF-κB) signal pathway [47]. However, the anticancer mechanisms in breast cancer cells and prostate cancer cells mainly occur through activating the AMPK/ULK1 pathway and inhibiting the expression of mTOR/Raptor complex 1 to cause autophagic cell death [48]. Quercetin is also a common flavonoid that is involved in reducing the risk of several types of cancer. A mutated Ras gene is found in various malignant tumors, especially colon cancer, leading to the occurrence and development of tumors. With the presence of oncogene Ha-Ras, quercetin-mediated autophagy increases the sensitivity of colon cancer cells [100,101]. Isoxanthohumol (IXN), which is a precursor to 8-prenylnaringenin (8-PN), is an important prenylflavonoid in hops and beer, in addition to xanthohumol (XN). Its pharmacological properties have gained notable attention in tumor chemoprevention [83,84]. In prostate PC3 and DU145, cell death resembling autophagic cell death is induced by IXN, which could not be rescued by caspase inhibitor benzyloxycarbonyl-Val-Ala-Asp-fluoromethyl ketone (zVAD-fmk) [83]. Additionally, IXN also induced autophagic and caspase-dependent apoptotic death in metastatic B16-F10 cells [84].

ROS production during oxidative stress is a crucial trigger of autophagy. Numerous signal pathways, such as PI3K/Akt/mTOR, Beclin-1, catalase, and the mitochondrial electron transport chain, have been shown to be involved [94]. Importantly, ACD-mediated caspase inhibition by the degradation of catalase further promotes the production of ROS [34]. Silibinin (SIL), which is the main flavonoid lignan extracted from milk thistle seeds, exhibits potent anti-tumor effects in many tumor models in vivo and in vitro, such as colon, prostate, and lung cancer [102]. Several studies indicate that silibinin promotes autophagic death through the ROS pathway in human fibrosarcoma HT1080, cervix carcinoma HeLa, and breast cancer MCF-7 cells [92,93,94,95]. Since H2O2 is the main factor of silibinin-induced ROS, reactive oxygen scavenger N-acetylcysteine (NAC) and H2O2 scavenger catalase reduced the cytotoxicity of silibinin, promoting cell survival by inhibiting autophagy and apoptosis [92,93,95]. Another study illustrated that the mechanism by which it induces p53-mediated autophagic death in HT1080 might depend on the activation of ROS-p38 and JNK MAPK pathways, as well as the inhibition of MEK/ERK and PI3K/Akt pathways. Moreover, the JNK signal pathway is not affected by ROS removal [93]. Reactive nitrogen species (RNS) represent another free radical related to ROS, which can induce apoptosis and autophagy through a common pathway with ROS. Silibinin’s growth inhibitory effect for HeLa cells is associated with the induction of ROS/RNS-mediated autophagy [95]. Bcl-2 adenovirus E1B 19-kDa-interacting protein 3 (BNIP3) is known as a potent inducer of mitochondrial autophagy in various tumor types accompanied by mitochondrial dysfunction. Silibinin-induced ACD in breast cancer cells was accompanied by ROS-dependent mitochondrial dysfunction caused by the alteration of ΔΨm and lack of ATP production that involved BNIP3 [94]. Additionally, silibinin and arsenic alone and in combination initiated autophagic cell death that increased the expression of the Beclin 1 protein by a higher level of ROS in the early stage of prostate cancer cell DU145 treatment [97]. Therefore, silibinin represents a potential effective inducer of autophagy through activation of the ROS pathway in various cell lines; however, its role needs to be explored further.

Hydroxysafflor yellow A (HSYA), which is one of the main bioactive and water-soluble compounds isolated from *Carthamus tinctorius* L., can function as a therapeutic agent for cardiovascular disorders and an effective anti-cancer natural product. A total of 2 µM HSYA resulted in cell autophagy in HepG2 cells, showing a chemoprevention property through significantly increasing the expression of Beclin 1, decreasing the phosphorylation of ERK. The survival rate of hepatoma cells treated with chloroquine (CQ)+ HSYA was increased, suggesting that HSYA may become a potential therapeutic drug for liver cancer by enhancing autophagy [91]. Nonetheless, there is insufficient research on the anti-tumor effect of HSYA through autophagy. The abnormal expression of the long non-coding RNA (lncRNA) prostate cancer gene expression marker 1 (PCGEM1) contributes to the occurrence of prostate cancer. LV3-shRNA PCGEM1 effectively increased the sensitivity of LNCaP cells to baicalein, and its molecular mechanism may be related to a decrease of the PCGEM1 expression and autophagy induction [53].

## 3. Interaction between Autophagy and Tumor Cell Migration, Invasion, and Angiogenesis

Metastasis is a unique characteristic of cancer cells and the severely serious stage of cancer, which is of great significance to clinical staging and prognosis. The process mainly involves epithelial-mesenchymal transformation (EMT), matrix metalloproteinases (MMPs) degradation of the extracellular matrix (ECM) to catalyze tumor metastasis, infiltration of the matrix, invasion of the vascular system, and the promotion of neovascularization [4]. Neovascularization, providing more nutrition for a solid tumor, is more conducive to tumor invasion into the circulatory system. A complex relationship exists between autophagy and cancer cell metastasis. Previous research has demonstrated that in some advanced metastatic diseases, such as advanced esophageal cancer, liver cancer, melanoma, glioblastoma, breast cancer, prostate cancer, myeloma, and so on, the upregulated expression of autophagy markers is associated with a poor prognosis [103]. Reversely, in gastric cancer cells, autophagy inhibition promotes EMT and metastasis, changes the metabolic phenotype from mitochondrial oxidative phosphorylation to aerobic glycolysis, and transforms the cell phenotype toward one that is malignant, which may further contribute to chemoresistance and a poor prognosis of gastric cancer [104].

**Figure 3:**
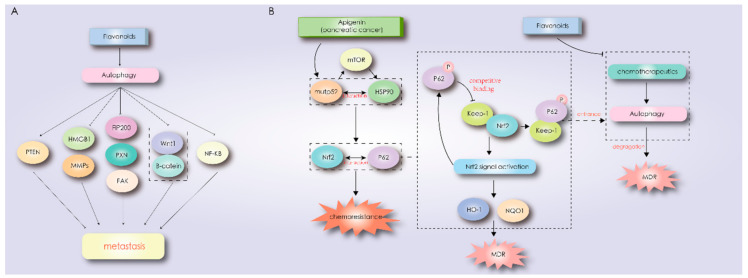
Flavonoids monitor autophagy affecting metastasis and multidrug resistance (MDR). (**A**) Flavonoid-induced autophagy mediates metastasis of tumor cells by the inhibition of the Wnt/β-catenin and NF-κB signal pathway and upregulation of the metastasis-related protein expression, and inhibits neovascularization via promotion of the tumor suppressor gene PTEN. (**B**) In PaCa44 cells, mutant p53 stabilizes through its interaction with HSP90, which can be upregulated by promoting mTOR activation, activating the positive feedback loop between Nrf2 and p62, and thereby inducing chemotherapy resistance to apigenin. In tumor cells, when autophagy is compromised, the competition between p62 accumulation and nuclear factor erythroid 2 (Nrf2) combination with keap-1 leads to the nuclear translocation of Nrf2 dissociation, promoting continuous activation of the Nrf2 signal pathway and p62 accumulation, while the p62/ kelch-like ECH-associated protein 1 (Keap-1) complex is isolated in autophagosomes and degraded by autophagy. Some effective chemotherapeutic drugs have been found to produce drug resistance by inducing the autophagy of tumor cells, yet flavonoids combined with these chemotherapeutic drugs can block this pathway through the inhibition of autophagy.

It has been reported that EMT-related signaling pathways influence the process of cell autophagy, such as integrin, WNTs, NF-κB, and transforming growth factor (TGF)-β signaling pathways, and autophagy activation reversely suppresses or enhances EMT by regulating different signal pathways, such as PI3K/AKT/mTOR, Beclin-1, p53, JAK/STAT, and so on [105]. Autophagy can either inhibit or promote tumor cell migration and invasion. On one hand, autophagy activation provides energy and fundamental nutrients for EMT in the process of metastasis and diffusion, while cells with EMT also need to activate autophagy subsidence to upregulate the cancer cell survival rate in the process of metastasis and diffusion [105,106]. Autophagy activation increases the metastasis-related protein expression, such as that of high-mobility group box 1 protein (HMGB1), metastasis-associated protein oncostatin M, and MMP-9; activates the TGF-β2/smad signal pathway to trigger the expression of EMT markers; and promotes the activation of β-catenin and Smad signals to regulate integrin-linked kinase (ILK) [105]. On the other hand, the metastasis-suppressive role of autophagy during the tumor cellular process involves selectively downregulating the key transcription factors or proteins of EMT in the early stage of tumorigenesis [105]. Autophagy caused by nutritional deprivation or inhibition of related mechanism targets of the mTOR pathway reduces the migration and invasion of glioblastoma cells [107]. Autophagy and cell motility at the molecular level are regulated by focal adhesion kinase (FAK), FAK interacting protein 200kD (FIP200), and Paxillin (PXN) [103]. Autophagy promotes cell movement through the degradation of plaque adhesion complexes, especially PXN. Integrin-mediated activation of the FAK-Src pathway inhibits autophagy and promotes E-cadherin-dependent cell movement and EMT [103]. Autophagy can also inhibit or promote tumor angiogenesis: The tumor suppressor gene PTEN inhibits neovascularization by inducing autophagy in mouse brain tumors, yet autophagy promotes the expression of the HMGB-1 protein, which is beneficial to angiogenesis and tumor cell survival in an anoxic microenvironment, as shown in Figure 3A [19,108,109]. At present, some flavonoids have been proven to induce the autophagy of tumor cells and affect cell migration and invasion.

In renal cell carcinoma (RCC), studies have indicated that silibinin decreased the invasion and migration of RCC 786-O cells in vitro, downregulating the expression of MMP-2 and MMP-9 and the urokinase plasminogen activator, and suppressing the MAPK or epidermal growth factor receptor (EGFR)/MMP-9 signaling pathway [110]. Li et al. first reported that silibinin triggered early autophagy and this may be involved in inhibition of the migration and invasion of RCC ACHN and 786-O cells by activating the AMPK/mTOR pathway in vitro [96]. Moreover, another study identified a novel mechanism where silibinin regulated the metastasis and EMT of RCC through the activation of autophagy, which resulted in the downregulation of Wnt/β-catenin signaling [110]. Interestingly, the invasion results of silibinin and silibinin + siRNA β-catenin groups displayed no statistical significance, possibly suggesting that different signaling pathways play different roles in the migration and invasion of cancer cells. However, it is still not clear how autophagy induction inhibits metastasis. Furthermore, silibinin strongly inhibited the tumorigenesis and metastasis of prostate cancer cells exposed to arsenic (0.5 or 5 µM), which repressed cell survival and invasiveness, mainly through the induction of autophagy and apoptosis. Silibinin also reduced the expression of MMP-2 and vimentin to limit cell motility and invasiveness, and regulated cell cycle-related proteins to induce cell arrest in the G1 or G2/M phase [97]. Adenoid cystic carcinoma (ACC), as the most common malignant tumor of epithelial salivary glands, is characterized by a unique neurotropic and high potential for lung metastasis associated with cancer progression, leading to a poor or fatal clinical prognosis [111]. A study revealed that the autophagy levels in patients with ACC were lower than those in normal glandular tissue, suggesting that autophagy induction might inhibit the proliferation of ACC cells and lung metastasis [98]. Another study by Jiang et al. revealed that the proliferation and lung metastasis of salivary gland adenoid cystic carcinoma (ACC-M) cells in nude mice might also be restricted by silibinin via modulating autophagic-associated proteins [98]. Additionally, in this study, when ACC-M cells were injected into the tail vein of mice for 24 h, that is, in the early stage of the tumor, the administration of silibinin triggered autophagy, which plays a critical role in tumor development. When the tumor develops to a certain stage, whether silibinin intervention still inhibits ACC cells or plays a promoting role remains to be explored further [98].

Xanthoangelol (XAG) is a major chalcone constituent isolated from *Angelica keiskei* Koidzumi with known antioxidant and antitumor effects against several types of cancer. In recent years, XAG has been reported to possess anti-hepatoma effects. XAG treatment of hepatocellular carcinoma (HCC) cells Hep3B and HuH7 did not reduce these cell viabilities instead of significantly inhibiting the ability of cell invasion, migration, and EMT. Yang et al. showed that autophagy induction via regulation of the AMPK/mTOR signal pathway reduced HCC cell metastasis and restored the lung tissue architecture. Therefore, autophagy activation was necessary for the anti-metastatic effect of XAG against HCC [82]. Isoliquiritigenin (ISL) is a kind of chalcone compound derived from licorice. A series of preliminary experiments have suggested that ISL may inhibit the proliferation, migration, and invasion of MKN28 gastric cancer cells with PI3K/AKT/mTOR signaling pathway-mediated apoptosis and autophagy [90]. IXN manifested its antitumor potential by inducing combined cell death through autophagy, as well as caspase-dependent apoptosis. IXN-diminished metastatic potential is due to disrupted integrin signaling and decreased cell invasion and migration-related proteins in vitro, including integrin α-6, FAK, vinculin, Rho kinase, α-smooth muscle actin, and β-Actin. In vivo, IXN decreased HMGB1 expression and enhanced the expression of the S100 protein, accompanied by the acquisition of a spindle shape and a low expression of proliferative marker Ki-67, which ultimately inhibited lung metastasis in a mouse model [84]. However, the intricate relationship between the integrin signaling pathway and autophagic cell death induced by IXN is still confused. Quercetin disturbed the migration and invasion and promoted the autophagy of hepatocellular carcinoma LM3 cells, partly relying on the suppression of JAK2 and STAT3 activation [69]. It has been suggested that autophagy reduces the level of glycolysis by regulating hexokinase 2 in hepatocellular carcinoma cells, while the inhibition of glycolysis blocks the energy supply of cell movement. In vivo and in vitro experiments confirmed that quercetin downregulated the expression of cell migration marker proteins and inhibited the glycolysis process by decreasing the acidity of the tumor microenvironment, which were induced by Akt-mTOR pathway-mediated autophagy [62].

## 4. A Complex Landscape in Autophagy, the Cell Cycle, and Senescence

Under adverse or stressful conditions, cells block the cell cycle temporarily or irreversibly, contributing to the regulation of proliferation during development and differentiation and preventing the spread of potential harmful cells [112]. Cell cycle progression is mutually regulated by cyclin, cyclin-dependent kinase (CDK), and the cyclin-dependent kinase inhibitor (CKI). It was proved that basal autophagy was detected in interphase (G0, G1, S, and G2) and mitotic (M) phases, while the level of autophagy in the M phase was significantly lower than that in the interphase [113]. There is an intricate interaction between autophagy and the cell cycle in the process of cell division. Some cell cycle regulators, including CDKs, cyclin, CKIs, the Aurora A enzyme, polo-like kinases (PLKs), p53, etc., are directly associated with autophagy or its related pathways, with cell cycle arrest caused by metabolic stress [112,113]. Autophagy is affected by regulation of the G1/S phase protein, G2/M phase protein, and M phase protein: CDK4 and CDK6 regulate the PI3K/AKT-AMPK signal pathway to inhibit autophagy; CDK2, CDKL3, and Aurora A inhibit the autophagy of many kinds of cancer cells by activating the activity of mTOR, resulting in cell death or a higher chemosensitivity; and the activation of p53 and CKIs (p16, p21, and p27) was observed at multiple checkpoints of the cell cycle, which induce autophagy and control tumor cell death by regulating specific CDK-cyclin complexes [112,113]. Moreover, compared with normal cells, the increased basic and/or stress levels of DNA repair and autophagy observed in tumor cells have been identified as the most important drug response procedures affecting the outcome of anticancer therapy. Furthermore, in tumor cells with inactivated cell cycle checkpoints, autophagy limits the accumulation of genomic damage and gene mutations, which reflects the role of autophagy in protecting the genome in the mechanism of tumor cell growth [114].

Generally, an abnormal autophagy function includes the removal of damaged mitochondria and the suppression of oxidative stress, DNA damage, telomere shortening, and oncogenic stress-triggered cell senescence [115]. However, the relationship between senescence and autophagy in tumor cells is profoundly subtle. Senescence and autophagy may be simultaneous or independent [113,115], for example, the long-term inhibition of autophagy does not affect the progress of senescence. Young et al. showed that the inhibition of autophagy delays senescence, but that this inhibition cannot completely prevent the oncogene-mediated senescence process [116]. Additionally, autophagy potentially initiated by oncogene-induced senescence produces a large number of amino acids to promote the secretion of aging factor senescence-associated secretory phenotype (SASP) [115]. Cytostatic autophagy is believed to inhibit cell proliferation in a form independent of apoptosis, differing from cytotoxic autophagy not leading to the induction of cell death, which is potentially associated with senescence [117]. Therefore, it remains to be seen whether autophagy has a positive or negative effect on senescence. Flavonoids, as natural inductors of premature senescence, are involved in the treatment and prevention of cancer. Sufficient studies have indicated that polyphenols can target the tumor microenvironment to prevent cancer via inhibiting the secretion of the SASP factor by senescent cells, while once cancer occurs, polyphenols regulate oncogenes, oxidative stress, the DNA damage response (DRR), ER stress, or autophagy to suppress tumors by inducing cancer cell senescence [118].

Ruela-de-Sousa et al. first reported the effect of apigenin on autophagy in cancer cells. They found that apigenin could induce autophagy and G0/G1 phase arrest in erythroid leukemia TF1 cells, while apoptosis and G2/M phase arrest were observed in myeloid HL60 cells. Significant downregulation of the expression of autophagy inhibitor mTOR and its downstream target p70S6K was noticed during apigenin treatment. Moreover, the activity of mTOR related to the autophagy pathway decreased in the cells of G0/G1 phase arrest. These results suggest that, at least in some types of leukemia, apigenin-induced autophagy by inhibiting mTOR activity is closely related to cell cycle arrest [119]. Apigenin also induced the G2/M phase arrest of thyroid papillary cancer cells, which led to autophagic death [37], although the specific mechanism was not elucidated. *Rhus coriaria* ethanolic extract (RCE), in which 211 phytochemicals have been identified, including flavonoids, isoflavonoids, and other compounds, can induce senescence and autophagic cell death in MDA-MB-231 cells though p38 and ERK1/2 activation [120]. As the mutant p53 level decreases, DNA damage is an earlier event in RCE-treated cells, which may serve as a trigger for downstream responses contributing to autophagy, senescence, and cell death. p21 plays an important role in cell cycle arrest and early senescence. It is upregulated in senescent cells and its overexpression is able to generate cell cycle arrest and premature senescence of breast cancer cells. Lower concentrations of RCE treatment can lead to cell senescence by inducing apoptosis-independent autophagy, while at higher concentrations, with the downregulation of p21, the proportion of aging cells decreases and cytotoxic autophagy occurs (the proportion of autophagic cell death increases). The downregulation of p21 may be used as a trigger to induce the autophagic death of MDA-MB-231 cells, which determines the type of cell death [120]. Several studies have shown that ERK1/2 is responsible for regulating the maturation of autophagosomes [120,121,122]. Similarly, in MCF-7 cells, low concentrations of Diosmin (5 and 10 μM) activated cytostatic autophagy by p53 and p21 upregulation, ERK activation, DNA damage, oxidative stress, and changes in global DNA methylation patterns, to induce senescence and permanent cell cycle arrest, while inducing cytotoxic autophagy accompanied by nitrosative stress at a higher concentration (20 μM) [121]. In contrast, the downregulation of p21 is not involved in the transition from cytostatic autophagy to cytotoxic autophagy. Moreover, in MDA-MB-231 and SK-BR-3 cells with no mutant p53 and DNA damage, cytotoxic autophagy has not been found. Therefore, p21, ERK1/2, p53, and the degree of DNA damage may determine the response of stress cells. Additionally, chalcone compound flavokawain B prompted the senescence of glioma cells by inducing ER stress-dependent autophagy, in which DNA double breakage was involved. On the contrary, the inhibition of autophagy promoted apoptotic cells and reduced senescent cells [86]. In summary, it can be speculated that flavonoids induce the cytostatic autophagy and senescence of tumor cells, which may benefit from their low cytotoxicity to activate the survival mechanism of tumor cells.

At present, experimental studies on the complex relationship between autophagy and the cell cycle or senescence induced by flavonoids seem incomprehensible, which makes this longstanding topic have greater development value.

## 5. Crosstalk between Autophagy and Apoptosis

Autophagy and apoptosis are two different forms of programmed cell death, which control two important physiological activities of cell survival and cell death. The significant differences in their shape and function do not affect the crosstalk and interrelation between them, which can be divided into three kinds: Synergism; antagonism; and mutual transformation. Flavonoids induce the autophagy and apoptosis of many tumor cells, yet the close relationship between autophagy and the apoptosis signal pathway makes the anti-tumor mechanism of flavonoids more complex. Therefore, understanding the interaction is helpful for targeting tumor-related therapy.

Autophagy and apoptosis interact through many regulatory factors and signal pathways, such as Beclin-1/Bcl-2; Fas-associated death domain-like IL-1β-converting enzyme inhibitory protein (FLIP); ATGs; caspase enzymes; ROS; and p53, JNK, and PI3K/Akt/mTOR signaling pathways [123,124]. Beclin-1 has been proven to be a BH3-only protein that can bind to the anti-apoptotic protein Bcl-2, directly regulating autophagy and apoptosis. Other BH3-only pro-apoptotic proteins (Bad, BNIP3, Puma, and BH3 domain mimics) also competitively combine with Bcl-2, which promotes Beclin-1 release and autophagy signal activation [124]. Interestingly, activated caspase enzymes are involved in the regulation of autophagy through interaction and cleavage with autophagy-related proteins. Activated caspase-3 and caspase-8, by decomposing autophagy-related proteins, inhibit autophagy and terminate the self-protection function of cells. Caspase-9, by interacting with ATG7, increases the lipids of LC3. Contrarily, the combination of caspase-9 and ATG7 inhibits the translocation of caspase-9 to apoptotic bodies and prevents apoptosis [123,125]. Anti-apoptotic protein FLIP competitively interacts with ATG3, leading to autophagy inhibition with downregulation of the liposuction of LC3, while autophagy activation conversely inhibits the effect between FLIP and ATG3 [124,126]. Therefore, ATGs (ATG4D, ATG12, ATG5, ATG3, and so on) have an important influence on apoptosis other than autophagy [125]. Mitochondrial damage, in addition to inducing apoptosis, can activate autophagy-related factors, initiating autophagy to capture and degrade damaged mitochondria. Therefore, the occurrence of mitochondrial autophagy may curb the process of apoptosis [124].

The tumor suppressor gene wtp53 is usually located in the cytoplasm and translocated to the nucleus after being phosphorylated directly or indirectly by kinases during stress [123]. Wtp53 may upregulate the transcription of pro-apoptotic genes (Bax, NOXA, Bim, and PUMA), and inhibit the transcription of anti-apoptotic gene Bcl-2 [127]. Nuclear p53 could transcriptively activate the damage-regulated autophagy modulator (DRAM) to enhance autophagy, while cytoplasmic p53 was observed to have the opposite role, inhibiting autophagy by regulating the FIP200 or AMPK/mTOR pathway [128]. P53 mutates in 50% of human cancers, so it cannot effectively activate its downstream DNA damage stress response pathway, and then loses its tumor inhibitory function. However, the interaction with heat shock proteins (HSP90, HSP40, and HSP1) prevents mutp53 from proteolytic degradation. Natural compounds have been proven to activate autophagy to produce degradation mutp53 and restore tumor chemosensitivity via stimulating endoplasmic reticulum stress to reduce the expression of HSP90, acting on protein folding, or reactivating wtp53 and its target gene DRAM [129]. Endoplasmic reticulum (ER) stress (or Ca^2+^) is a mixed stimulator of apoptosis and autophagy. The preceding study indicated that, after ER stress, the released Ca^2+^ caused the expression of calcium/calmodulin-dependent protein kinase β and activated the AMPK/mTOR pathway, contributing to autophagy and apoptosis [130].

### 5.1. Synergism between Autophagy and Apoptosis

Lv et al. showed that isovitexin inhibited the proliferation of HCC cell Hepg2 and SK-Hep1 and induced autophagy and apoptosis through depending on ER stress. Moreover, the inhibition of autophagy reduced the number of isovitexin-induced apoptotic cells [46]. Cytoplasmic Ca^2+^ overload not only induced autophagy through the AMPK/mTOR pathway, but also promoted ER stress and cell apoptosis. This may be the reason why baicalin exhibits a cytotoxic effect on human glioblastoma cells [55]. Several studies in vivo and in vitro have also reported that quercetin promoted apoptotic HCC cells through autophagy stimulated by modulating both AKT/mTOR and the MAPK pathway [63], and quercetin-induced autophagy flux also enhanced the tumor necrosis factor (TNF)-related apoptosis-inducing ligand (TRAIL)sensitivity in A549 human lung cancer cells [131]. TGF-β1 exerts antitumorigenic functions mediated by Smad3, which is a main downstream effector of the TGF-β signaling cascade during the early stage of carcinogenesis. Galangin effectively suppressed the phosphorylation of Thr-179 in the Smad3 linker region by reducing the phosphorylation of CDK4, resulting in an enhanced TGF-β1 signal to promote apoptosis and the growth inhibition of cancer cells (prostate cancer and pancreatic cancer) [132]. Furthermore, it has been shown that galangin induced autophagic death in Hepg2 cells dependent on the activation of the TGF-β1/Smad and AMPK/SIRT1 pathway, which also intensified apoptotic death [133,134,135]. Silent information regulator 2 homolog 1 (SIRT1), as an NAD-dependent class III histone deacetylase, has an important role in autophagy. In HepG2 cells, galangin enhanced the binding of SIRT1-LC3 and reduced the acetylation of endogenous LC3 by upregulating SIRT1 expression to induce autophagy [135]. All in all, in hepatocellular carcinoma cells, TGF-β1/Smad, AMPK, p53 pathway activation, and LC3 deacetylation were identified to display a close relationship with SIRT1 for galangin-induced autophagy [133,134,135].

### 5.2. Antagonism Amid Autophagy and Apoptosis

Numerous studies have demonstrated that flavonoids, such as apigenin, baicalin, isoxanthohumol, and quercetin, can induce apoptosis and autophagy in breast cancer, human colon cancer, neuroblastoma, liver cancer, oral squamous cell carcinoma, ovarian cancer, melanoma, and other cancer cells, in which autophagy executes a protective effect of tumor cell survival [38,39,40,41,56,58,59,76]. Therefore, the combined application of flavonoids and autophagy inhibitors may be a promising strategy for controlling the occurrence and development of tumors. Although flavonoids induce protective autophagy, the same compound has different molecular pathways and multiple autophagic targets for different types of cancer or cell lines of the same cancer. Baicalin induced ROS-dependent autophagy in oral squamous cell carcinoma and induced autophagy by activating ER stress, inhibiting the AKT/mTOR pathway in hepatocellular carcinoma [56,57,59]. The activation of ERK is also involved in the protective autophagy of ovarian cancer cells HEY and A2780 with baicalein administration. After pretreatment with ERK inhibitor U0126 or siRNA ERK, LC3-II, which is a marker of autophagy induced by baicalein, was significantly inhibited [58]. Autophagy triggered by isoxanthohumol in melanoma cells played a cytoprotective role and did not mediate cytotoxicity [85].

Quercetin-induced ER stress, as a “double edge sword”, activated protective autophagy and apoptosis via the p-STAT3/Bcl-2 axis in ovarian cancer. Unexpectedly, the inhibition of ER stress did not reverse quercetin-induced cell death, indicating that ER stress is not the only pathway inducing apoptosis in ovarian cancer cells [70]. The use of quercetin and resveratrol could attenuate quercetin-induced protective autophagy, downregulate resveratrol-induced ER stress, and enhance non-ER stress-related apoptosis. The mechanism of this combination in autophagy and apoptosis may be related to the regulation of AMPK phosphorylation, heme oxygenase 1 (HO-1), lysosomal membrane permeabilization (LMP), and Zinc (Zn^2+^) dynamics [64]. In addition, apoptosis and protective autophagy were also noticed in HL-60 acute myeloid leukemia (AML) cell death mediated by quercetin due to caspase activation [136,137]. Under a high level of ER stress, the activation of Epigallocatechin-3-Gallate (EGCG)-mediated autophagy served as a cytoprotective player by affecting the balance of mTOR-AMPK, thus postponing apoptotic cell death by promoting autophagy upon ER stress [138].

PI3K/AKT/mTOR, Wnt/β-catenin, and STAT3 also participated in quercetin-induced cell death in primary effusion lymphoma (PEL) [71]. Additionally, many articles on quercetin have revealed that apoptosis and autophagy induction was regarded as the main explanation for the cytotoxic effect. Quercetin caused protective autophagy and apoptosis in gastric cancer cells by blocking the phosphorylation of Akt, mTOR, and downstream of p70 S6K (S424/T421) and 4E-BP1 (S65/T70), accumulating hypoxia-induced factor 1α (HIF-1α), which enhanced BNIP3/BNIP3L to disrupt the interaction of Beclin 1 with Bcl-2/Bcl-xL [73].

Hsp72 confers protection against apoptosis and mediates survival in response to cell stress by inducing autophagy. In a rat model of lipopolysaccharide (LPS)-associated peritonitis, the overexpression of HSP72 mediated JNK-dependent autophagy, which might be repressed by quercetin [139]. When anaplastic astrocytoma MOGGCCM cells were incubated with quercetin and temozolomide, autophagic cell death was decreased with the increasing concentrations of quercetin, accompanied by a high level of necrosis and apoptosis. The mechanism for this is as follows: Quercetin reduced autophagic cell death by inhibiting the expression of HSPs, resulting in increased apoptosis [66]. Likewise, silencing the expression of HSP27 and HSP72 in glioblastoma multiforme T98G and anaplastic astrocytoma MOGGCCM cells makes them extremely vulnerable to apoptosis induced by temozolomide and/or quercetin, and soluble epoxide hydrolase inhibitor (sEHi) trans-4[-4-(3-adamantan-1-yl-ureido)-cyclohexyloxy]-benzoic acid (t-AUCB)-induced autophagy in glioblastoma U251 and U87 cells was also blocked by the inhibition of Hsp27 with quercetin [65,67]. Sorafenib administered with quercetin also has the same influence on T98G and MOGGCCM cells. It is a very effective inducer of programmed cell death, especially in cells inhibiting heat shock protein expression [68]. HSPs can stabilize several client proteins associated with cell survival, including c-Myc. The activation of PI3K/AKT/mTOR stabilizes the expression of c-Myc by upregulating HSP70, while c-Myc positively regulates the activity of mTOR to promote the survival of Burkitt’s lymphoma cells. In accordance, quercetin treatment induced autophagy, possibly through inhibiting the PI3K/AKT/mTOR pathway and partially degrading the mutant c-Myc protein [72].

Generally, tumor suppressor protein p53 promotes autophagy, whereas mutant p53 silences this process [140]. Galangin induced autophagy through the activation and overexpression of p53 in hepg2 cells [77]. Moreover, in pancreatic cancer Panc1 cells, apigenin-incurred autophagy leads to mutp53 degradation, which inhibits autophagy by inducing mTOR activation, but it has different mechanisms in PaCa44 cells [42]. Apigenin also induced pancreatic cancer cell apoptosis harboring the p53 mutation by reactivating wtp53 [141]. In human glioblastoma U373MG cells, quercetin induced protective autophagy and apoptosis, accompanied by activating JNK and increasing and translocating mutp53 to mitochondria, but it is not clear whether quercetin restrains autophagy and promotes apoptosis through increased cytoplasmic p53 translocation to mitochondria [74].

### 5.3. Mutual Transformation in Autophagy and Apoptosis

Protective autophagy of human glioblastoma U373 MG cells was induced by quercetin [74]. Under the condition of nutritional starvation-induced autophagy simulating the tumor microenvironment, T98G glioma cells were treated with quercetin for 24 h. It was found that quercetin inhibited the occurrence of hunger-induced protective autophagy and promoted apoptosis. The results are inconsistent with a previous study, potentially due to the inconsistency of experimental conditions [74,142]. In addition, luteolin inhibited rapamycin induced autophagy and promoted apoptosis in glioblastoma cells and animal models, and a combination of luteolin and silibinin through the overexpression of miR-7-1-3p dramatically blocked the occurrence of autophagic populations [143]. The inhibition of protective autophagy generally enhances the therapeutic effect of flavonoids on tumors. However, the inhibition of different stages in the process of autophagy may lead to an inconsistent effect of flavonoids on tumor treatment. Compared with 3-MA for the early inhibition of autophagy, which attenuated the cytotoxicity induced by imatinib, the autophagy inhibition of baffinomycin A1 in the late stage enhanced imatinib-induced cytotoxicity [144]. In addition, autophagy inhibition in the late stage (inhibitor CQ) rather than in the early stage (inhibitor 3-MA or shRNA Beclin1) also enhanced the therapeutic effect of malignant gliomas with quercetin [145]. The reason for this may be that CQ leads to the accumulation of autophagy vacuoles and induces caspase-3-dependent apoptosis.

### 5.4. Others

A large number of flavonoids have the ability to induce tumor cell apoptosis and autophagy, but the interaction between them is not clear. Baicalein inhibited the proliferation of MCF-7 and MDA-MB-231 breast cancer cells, Fro undifferentiated thyroid cancer cells, and MGC-803 gastric cancer cells, and induced apoptosis and autophagy. It downregulated the expression of downstream proteins phosphorylated Akt (p-AKT), phosphorylated mTOR (p-mTOR), NF-κB, and phosphorylation of inhibitor of kappa B (p-IκB), which play a regulatory role in autophagy, apoptosis, and tumor growth [50,51,146]. Drp1-dependent mitochondrial fission is an early and key event of apoptosis, while excessive division will lead to mitochondrial dysfunction, apoptosis, and autophagy. Deng et al. found that Drp1-mediated mitochondrial division exerts an anti-tumor effect by activating the AMPK pathway, causing it to participate in baicalein-induced apoptosis and autophagy of lung cancer cells [52]. Baicalein also induced autophagy and apoptosis by activating the AMPK pathway in glioma cells [147]. Licochalcone A (LA) and galangin have been proven to increase autophagy and apoptosis in cancer cells via the inhibition of PI3K/Akt/mTOR activation and downregulation of Bcl-2 expression [78,87]. Furthermore, in LNCaP prostate cancer cells treated with 12.5 μM LA, the degree of cell death was more obvious than that of apoptosis, indicating that apoptosis may not be the only mechanism of LA-induced cell death, and may also induce autophagy-related cell death [148]. In human neuroglioma cells, quercetin nanoparticles activated AKT/ERK/Caspase-3 to induce autophagy and apoptosis [149], while in cervical cancer cells, they inhibited the JAK2 signaling pathway to induce apoptotic and autophagic cell death [150].

## 6. Targeting Autophagy Regulates MDR

The multidrug resistance (MDR) of tumor cells is the main motivation for the failure of clinical treatment of tumors. Its mechanism is complex, dynamic, and multi-factorial, causing a difficult problem in tumor chemotherapy. The common mechanisms of drug resistance in MDR include ATP-binding cassette (ABC) transporter overexpression, inhibition of the apoptosis signal pathway, an imbalance of the DNA damage/repair process, epigenetic variation, microRNA mutations, tumor stem cell involvement, drug target modification, and autophagy [151,152]. It has been reported that the drug resistance of many kinds of cancer cells (colorectal cancer [153], esophageal cancer [154], prostate cancer [155], leukemia [156], etc.) increases with the increase of autophagy activity, while the inhibition of autophagy can restore drug-resistant cancer cells’ sensitivity and enhance the efficacy of chemotherapy. However, its continuous activation leads to autophagic cell death that may overcome MDR. In short, autophagy also has dual effects on tumor drug resistance promotion or inhibition [20].

As a mechanism for survival, autophagy promotes the development of multidrug resistance. For example, chemotherapeutic drugs participate in the regulation of autophagy through regulatory factors and signal pathways, in order to help tumor cells escape their killing effects and produce MDR [154,155,156]. Insufficient apoptosis is considered to be one of the main factors of multidrug resistance [157], and the activation of protective autophagy mentioned above can suppress apoptosis and further promote MDR. Studies have found that the HMGB1 protein is highly expressed in diverse solid tumors, mainly through the regulation of autophagy in drug-resistant tumor cells, playing an anti-apoptotic role. Liu, Yang et al. demonstrated that chemotherapeutic drugs (vincristine and doxorubicin) induced human leukemia cells to release HMGB1, and activated autophagy through the PI3KC3/MEK/ERK signaling pathway to remove abnormal proteins, organelles, or ROS, in order to promote the drug resistance of tumor cells [156,158]. Moreover, excessive autophagy of tumor cells blocks the occurrence of MDR. The inhibition of autophagy re-sensitizes drug-resistant cancer cells and enhances the efficacy of chemotherapeutic drugs. Autophagic cell death may be a solution for inducing apoptosis-tolerant tumor cell death.

As an essential regulator of multidrug resistance, microRNAs (miRNAs) can indirectly promote or inhibit autophagy by targeting various target genes or proteins and affect the sensitivity of drug-resistant tumor cells to chemotherapeutic drugs [159,160,161,162]. In addition, regulatory factors such as ATG gene silencing, the p53 gene, the ABC transporter, and the epidermal growth factor receptor (EGFR), and signal pathways such as MAPK, PI3K/Akt/mTOR, and ERK/NF-κB, also regulate autophagy participating in the multidrug resistance process [151,163]. However, the dual mechanism of autophagy on MDR is still controversial and requires further research. In clinical studies, it has been revealed that flavonoids in a variety of diets can increase the chemosensitivity of drug-resistant tumor cells through mediating the autophagy process. Flavonoids as multi-target antineoplastic drugs, combined with chemotherapy drugs, can avoid the occurrence of feedback loops that may appear when using a single target drug, subsequently inhibiting the induction of MDR.

ATG7 is a potential target of miR-520b, and Gao et al. showed that an apigenin-induced expression of miR-520b in drug-resistant hepatoma cells inhibited ATG7-dependent autophagy and significantly increased the sensitivity of BEL-7402/ADM-resistant cells to doxorubicin. These findings confirmed apigenin as a potential chemical sensitizer for hepatocellular carcinoma [36]. For the proliferation inhibition of drug-resistant colon cancer cells, apigenin inhibited the PI3K/AKT/m-TOR signal pathway of cisplatin-resistant colon cancer cells in vivo and in vitro, and induced autophagy to suppress cell growth [43]. Abnormal glucose uptake is a metabolic feature of tumor cells, which provides the energy required to support cell proliferation, metastasis, and drug-resistant survival. The combination of apigenin and gefitinib has inhibitory synergistic effects on tumor growth and can contribute to the proportion of apoptosis by inhibiting the AMPK signal pathway, impairing glucose utilization, and blocking autophagy flow in drug-resistant NSCLC cells [44].

Continuous activation of the nuclear factor erythroid 2 (Nrf2) signaling pathway can lead to tissue injury, inflammation, tumorigenesis, and chemotherapy resistance [164]. The phosphorylation of p62 correlates autophagy with the Nrf2/kelch-like ECH-associated protein 1 (Keap-1) signaling pathway. When autophagy is compromised, the competition between p62 accumulation and Nrf2 combination with keap-1 leads to the nuclear translocation of Nrf2 dissociation, promoting the continuous activation of the Nrf2 signal pathway and p62 accumulation, while the p62-Keap-1 complex is isolated in autophagosomes and degraded by autophagy [165]. Specifically, unlike pancreatic cancer PANC1 cells, in PaCa44 cells, mutant p53 stabilizes through its interaction with HSP90, activating the positive feedback loop between Nrf2 and p62, and thereby inducing chemotherapy resistance to apigenin, as shown in Figure 3B [42].

Vitexin is a kind of c-glycosylated flavonoid, existing in various medicinal plants. Vitexin inhibited autophagy by reducing the expression of autophagy marker proteins ATG5 and beclin1 and the transformation of LC3-I to LC3-II, so as to enhance the apoptotic response and reduce the drug resistance of multidrug-resistant cell line HCT-116^DR^. MDR-1 is an important protein related to cell drug resistance. Vitexin aggravated this response by inhibiting the expression of MDR-1 [45]. Lin et al. firstly found that isoliquiritigenin combined with a specific concentration of doxorubicin has a synergistic cytotoxic effect on multidrug-resistant human uterine sarcoma cell line MES-SA/Dx5, but it is more effective on MES-SA/Dx5-R cells with a high drug resistance. ISL regulated the expression of Bcl-2, the mTOR pathway, and the expression of other related proteins, triggering apoptosis and autophagy in drug-resistant tumor cells, while the inhibition of autophagy enhanced ISL-mediated drug-resistant cell death [88]. Some studies have shown that the resistance of human glioblastoma cells (GBM8901) to temozolomide (TMZ) is associated with the expression of O6-methylguanine-DNA-methyltransferase (MGMT), while TMZ-induced autophagy depends on the expression of MGMT [60]. Liao et al. demonstrated that chrysin overcomes the drug resistance of glioblastoma cells to TMZ by reducing the formation of LC3-II, the ATG12-ATG5 conjugate, and the expression of ATG7 and BECLIN1, in order to inhibit TMZ-induced autophagy and downregulate the expression of MGMT. In addition, it was also confirmed that chrysin inhibited the autophagy flux of these drug-resistant cells through an mTOR-independent pathway [60,61]. Although inducing protective autophagy in diverse tumor cells, EGCG alleviated gefitinib resistance in NSCLC by inhibiting autophagy and augmenting cell death through the inhibition of ERK phosphorylation [81].

Baicalein also reversed drug resistance by inhibiting autophagy [49]. The overexpressed mTOR gene in malignant liver tumors upregulates CD133 and promotes the cytochemical resistance, which means that clinical trials show a poor prognosis. A chemically resistant CD133(+) tumor-initiating stem cell-like cell (TIC) was isolated from mouse and human liver tumors. Baicalein treatment inhibited autophagy induced by the mTORC1 inhibitor tesirolimus (CCI-779), which synergistically induced cell death caused by mTORC1 inhibition in TIC and Huh7 cells and an HCC xenotransplantation model. The reason for this may be that baicalein blocks autophagy stimulated by mTORC1 inhibitors by interfering with the GTP binding activity of SAR1B GTPase, suggesting that baicalein targeted SAR1B to regulate autophagy to solve the drug resistance of TIC [54].

On the other hand, flavonoids also trigger the autophagy of drug-resistant tumor cells to resist MDR. As an autophagy inducer, isoliquiritigenin enhanced the chemosensitivity of drug-resistant cells. ISL aggravated the autophagy of drug-resistant breast cancer cells, accompanied by the checkpoint block of G2/M and downregulation of ABCG2 expression, but not including apoptosis. It promotes the degradation of ABCG2 and the influx of chemotherapeutic drugs through the autophagy-lysosome pathway. This study also confirmed that ULK1, as an early regulator of autophagy induction, was directly targeted by ISL-mediated miR-25 that led to autophagic death, improving the chemosensitivity of breast cancer [89]. P-gp located on the cell membrane, like an efflux pump, is responsible for reducing intracellular drug accumulation and mediating MDR. After the treatment of BEL-7402/5-FU cells with baicalein, the multidrug resistance could be reversed by baicalein through inducing autophagy in a concentration-dependent manner. The mechanism may be related to the downregulation of P-gp and Bcl-xl expression [49]. The receptor for advanced glycation end products (RAGE) is highly expressed in diverse tumor cells, acting on the inflammatory pathway to promote the development of tumors. It has been demonstrated that quercetin attenuated RAGE expression to facilitate cell autophagy and apoptosis, as well as the sensitivity of MIA PACA-2 and drug-resistant MIA PACA-2GEMR cells to gemcitabine [75].

These studies have proved that flavonoids are natural autophagy inducers that can improve the chemosensitivity of tumor cells. They induce autophagy through various pathways that lead to the death of drug-resistant tumor cells or increase the sensitivity of drug-resistant cells to chemotherapeutic drugs.

## 7. Clinical Trials

Apigenin, quercetin, EGCG, and curcumin have exhibited potential clinical benefits in various diseases, and have served as anticancer agents in clinical research to explore their pharmacological mechanism and toxicology. An intravenous bolus injection was confirmed as a safe mode of administration in a phase I clinical trial for evaluating the safety profile of quercetin [166]. Although the absorption of oral administration is limited, it shows biological activity in some patients with cancer. In an ongoing clinical study, a bioflavonoid mixture (containing 20 mg apigenin and 20 mg EGCG) was employed as a daily nutritional supplement for patients with colorectal cancer to evaluate its preventive effect on neoplasia recurrence [167]. Several clinical studies have proved that curcumin is safe and effective in the treatment of human colon cancer, colorectal cancer, benign prostatic hyperplasia and prostate cancer, pancreatic cancer, and breast cancer [168]. For healthy volunteers, the short-term administration dose of intravenous liposome curcumin was safe when the drug level peaked at 120 mg/m^2^ [168,169]. In a phase I trial, docetaxel plus curcumin was administered with an increase in dose in patients with advanced breast cancer, improving biological and clinical responses in most patients [169]. Meriva (curcumin plant) was tested and alleviated the side effects of cancer chemotherapy in a group of cancer patients (taking 1500 mg/day) for six weeks in a controlled semi-quantitative clinical study. The results achieved a better efficacy, improving the quality of life of the patients and inhibiting systemic inflammation [168,169]. As a chemopreventive agent, EGCG or green tea has been extensively examined in clinical trials for various cancers, such as pharyngeal, esophageal, gastric, prostate, oral, colon, pancreatic, liver, lung, and breast cancer [168]. In a pilot clinical study assessing the efficacy of EGCG capsules (400 mg/day in three divided doses for 2-8 weeks), involving 10 female patients (38-55 years old) with locally advanced non-inflammatory breast cancer receiving radiotherapy, the capsules were found to enlarge the efficacy of radiotherapy in breast cancer patients [168,169]. In regard to flavonoids, though many compounds have been reported to be therapeutic adjuvants ameliorating the side effects of chemotherapy or radiotherapy, the relative clinical trials lack explorations of the ability of flavonoids to either strengthen or overcome resistance to chemotherapeutic agents in cancer.

## 8. Conclusions

Abnormal autophagy has been shown to be related to the malignant phenotype and a poor prognosis of human cancer, but the detailed mechanism is still unclear. The role of autophagy in cancer is context-dependent, inhibiting the growth of cancer cells in the early stage of tumorigenesis and promoting tumor survival in advanced malignant tumors [34]. Understanding the dependence of autophagy on the cell type specificity will help to clarify the mechanism of autophagy in tumor cells. In most tumor cells, highly expressed autophagy-associated proteins and signaling pathways are activated to coordinate autophagy and promote tumor cell growth and survival. The drug inhibition of autophagy is expected to be an effective anticancer strategy. Some small autophagy inhibitors have been studied and used in the treatment of several diseases, such as PI3K inhibitors (3-MA, wortmannin, and LY294002), mTOR inhibitors (rapamycin and its analogues, RAD001, CCI-779, and Deforolimus), an autophagososome formation inhibitor (desmethylclomipramine), autophagolysosome formation inhibitors (CQ, bafilomycin A1, and thapsigargin), etc., but these inhibitors are well-tolerated and have limited or no curative effect [30]. All in all, the lack of a molecular target in these inhibitors and concerns about the adaptability of the system in the body underscore the need to develop more potent, specific, and translatable inhibitors of autophagy. Low-toxicity natural compounds (such as flavonoids), especially those of a dietary origin, are receiving increasing attention in the prevention and treatment of diseases, including cancer. Elucidating that flavonoid-induced autophagy promotes or inhibits cell survival may provide a new perspective for clinical research on flavonoids that may circumvent drug resistance by targeting protective autophagy [70].

The above summary shows that different flavonoids play different roles in various types of cancer cells, which can be regarded as autophagy inhibitors and autophagy inducers. Silibinin-induced autophagy has toxic effects on cells, such as inducing autophagic cell death, inhibiting cell invasion and migration, and overcoming drug resistance. Quercetin and EGCG mainly induce protective autophagy and play a protective role in promoting tumor growth, even in many human diseases. In addition, quercetin and luteolin also inhibit the occurrence of hunger-induced protective autophagy in glioblastoma. Chalcone compounds also exert an anti-tumor effect by targeting tumor cell autophagy; although there are not many studies at present, it has partly been shown that autophagy can be used as a therapeutic target of chalcone compounds in tumor cytochemotherapy. For example, isoliquiritigenin inhibits autophagy to enhance the sensitivity of drug-resistant uterine sarcoma cells to doxorubicin, and induces autophagy and epirubicin to promote the death of drug-resistant breast cancer cells; hydroxysafflor yellow A-mediated autophagy suppresses the proliferation of hepatoma cells; and XAG-triggered autophagy contributes to the anti-metastatic capacity of hepatoma cells. Interestingly, flavonoids overcome MDR by regulating autophagy induced by chemotherapeutic drugs, irrespective of whether they induce protective autophagy or autophagic cell death in non-drug-resistant cells. The possible reason for this is that the pathway of autophagy mediated by flavonoids is different from that of chemotherapeutic drugs. Taken together, the combined use of antineoplastic chemotherapeutic drugs and flavonoids will help to solve the common problem of multidrug resistance of chemotherapeutic drugs. Although it has been preliminarily confirmed in vivo and in vitro that the combination of flavonoids and chemotherapeutic drugs plays a role through targeted autophagy, clinical trial data are currently insufficient.

After different types of cells are treated with flavonoids, autophagy activated by the kinase signal pathway or transcription factors may be simultaneously involved in the regulation of cell growth and proliferation, apoptosis, necrosis, cell cycle arrest, senescence, cell migration, invasion, tumor angiogenesis, and multidrug resistance. In this paper, a total of 67 articles about the effect of flavonoids on autophagy were summarized, of which 33 were regulated by signal pathway PI3K/AKT/mTOR, as shown in Figure 2. This demonstrates the importance and universality of this signal pathway in affecting the autophagy of flavonoids. An abnormal expression of the PI3K/Akt gene regulates the exchange of autophagy activity. In the initial stage of autophagy, PI3K, AKT, AMPK, mTOR, ULK1, and beclin1 kinases are implicated in the regulation of autophagy, while flavonoids coordinate the expression of these kinases through ER stress, ROS, p53, Bcl-2, microRNA, HMGB1, glycolysis, and other pathways to affect autophagy. In this review, it was found that the current studies on flavonoid-mediated autophagy are mainly focused on the initial stage of autophagy membrane formation, while there are few studies on the late extension, maturation, and fusion stage, which may represent another direction of flavonoid targeted autophagy research. Flavonoids affect the occurrence and development of tumor cells by stimulating autophagy signals, so targeted autophagy may become the core approach of flavonoids in the treatment of tumors.

In summary, activation of the autophagy signal plays a vital role in the anti-tumor effect of flavonoids, especially the interaction with other signal pathways. However, the clinical application of flavonoids is limited due to their diverse structure, numerous binding sites, and complex action mechanisms. In addition, it is also necessary to strengthen in-depth research, such as structure–activity relationship research, so that flavonoids can be used as leading compounds for structural modification and structural optimization to develop a new generation of drugs.

## Figures and Tables

**Table 1:** Flavonoids in cancers with possible mechanisms discussed in this paper.

	Flavonoids	Cancer Model Cell Line/Animal	Mechanisms	Pharmacological Effects	Ref.
Flavones	Apigenin	Skin cancer SKH-1 mouse	Autophagy induced by AMPK-mTOR axis to prevent UV-mediated skin cancer	-	[6,7]
		Hepatocellular carcinoma doxorubicin-resistant cell BEL-7402/ADM Nude mice	Sensitizes drug-resistant cells to doxorubic through suppressing miR-520b/ATG7 axis	Autophagy promotes the occurrence of MDR	[36]
		Papillary thyroid carcinoma BCPAP cells	Induces autophagy by promoting beclin-1 expression, LC3 protein transformation, p62 degradation, and AVO accumulation Stimulates ROS production and DNA damage and downregulates Cdc25C expression, resulting in G2/M cell cycle arrest and autophagic death	Induction of autophagic cell death	[37]
		Breast cancer T47D, MDA-MB-231 Colorectal cancer HCT116 Hepatocellular carcinoma Hepg2 Male BALB/c nude mice Neuroblastoma SH-SY5Y, SK-N-BE2, IMR-32 (N-(4-hydroxyphenyl) retinamide + apigenin)	Increases levels of Caspase-3, PARP cleavage, and Bax/Bcl-2 ratios Augments the level of LC3-II and accumulation of AVOs Autophagy inhibitor significantly enhanced the apoptosis Induction of protective autophagy and apoptosis	Induction of protective autophagy and inhibition of apoptosis	[38,39,40,41]
		Pancreatic cancer Panc1, PaCa44	In Panc1 cells, apigenin slightly activated autophagy by suppression of mTORC1, downregulation of mutp53 and some of its nuclear output In PaCa44 cells, mutant p53 stabilizes through interactions with HSP90, activating the interaction between NRF2 and p62	-	[42]
		Colorectal cancer cisplatin-resistant cell HT-29 BALB/c nude mice	Induces autophagic cell death and inhibits the growth of cells by targeting m-TOR/PI3K/AKT signaling pathway	Autophagy inhibits the occurrence of MDR	[43]
		Non-small cell lung cancer EGFR-TKIs-resistant NCI-H1975 (Apigenin + Gefitinib)	Downregulates Cyclin D1, CDK4, E-cadherin, MMP2, and MMP9, and induces G0/G1 cell cycle arrest and cell metastasis Inhibits multiple oncogenic drivers such as c-Myc, HIF-1α, and EGFR, and reduces Gluts and MCT1 protein expression Inhibits the AMPK pathway and autophagy flux, leading to enhanced apoptotic cell death.	Inhibition of protective autophagy and induction of apoptosis	[44]
	Vitexin	Colorectal cancer Multidrug-resistant cell line HCT-116^DR^ BALB/c pathogen-free athymic nude mice	Decreases the expression of ATG5 and BECN1 and the transformation of LC3-I to LC3-II to inhibit autophagy, enhance apoptosis, and overcome MDR	Autophagy inhibition promotes apoptosis and overcomes MDR	[45]
	Isovitexin	Hepatocellular carcinoma Hepg2, SK-Hep1 Hepg2 tumor-bearing mouse	Induces apoptosis by promoting Bax, cleaved-Caspase-3, PARP expression, and the release of Cyto-c Stimulates the expression of autophagy-related proteins (LC3II, ATG3, ATG5, Beclin1) to induce autophagy Autophagy inhibition by silencing ATG5 or BFA reduces apoptosis, but activates caspase-3 Upregulates the expression of IRE1 α, XBP-1s, CHOP, and GRP-78 protein and induces ER stress Induces apoptosis and autophagy through ER stress	Induction of autophagy promotes apoptosis	[46]
	Baicalein	Thyroid carcinoma MDA-T68	Increases the concentration of apoptotic protein and the ratio of Bax/Bcl-2 Regulates NF-κB signal pathway to induce autophagic cell death Induces G2/M arrest by decreasing the concentration of Cyclin B1 protein	Induction of autophagic cell death	[47]
		Prostate cancer PC-3, DU145 Breast cancer MDA-MB-231	Upregulates the expression of Beclin1, Atg5, Atg7, ULK1, and LC3B-II Activation of AMPK and ULK1, and downregulation of mRNA level of mTOR/Raptor induces autophagic cell death	Induction of autophagic cell death	[48]
		Hepatocellular carcinoma 5-FU-resistant cell BEL-7402/5-FU	Induces apoptosis and autophagy and overcomes MDR. The mechanism may be related to the downregulation of P-gp and Bcl-xl expression.	Induction of autophagy to overcome MDR	[49]
		Breast cancer MCF-7, MDA-MB-231 Female BALB/c nude mice Thyroid cancer Fro	Induces apoptosis and autophagy by inhibiting PI3K/AKT pathway Downregulates the expression of p-AKT, p-mTOR, NF-κB, and p-IκB	-	[50,51]
		Non-small cell lung cancer A549, H1299 Male C57BL/6 mice	Induces the loss of mitochondrial membrane potential and the release of Cyto-c and apoptosis inducing factor into the cytoplasm Induces autophagy and activates autophagy flux In vivo and in vitro, DRP1-mediated mitochondrial division is involved in BA-induced apoptosis and autophagy by activating AMPK pathway	-	[52]
		Prostate cancer LNCaP	siRNA targeted for PCGEM1 increases sensitivity of LNCaP cells to baicalein	-	[53]
		Hepatocellular carcinoma TIC^DR^, Huh7 Male NSG^TM^ mice (NOD-SCID-Il2rg−/− mice) with HCC cells obtained from a patient	Inhibition of autophagy induced by CCI-779 Synergistic induction of cell death caused by mTORC1 inhibition in cell and HCC xenotransplantation models with CCI-779 Blocks autophagy stimulated by mTORC1 inhibitors by interfering with the GTP binding activity of SAR1B GTPase	Inhibition of autophagy and overcoming MDR	[54]
		Glioblastoma U87, U251 Male BALB/c athymic nude mice	Induces mitochondrial apoptosis Activation of autophagy through PI3K/Akt/mTOR pathway Inhibition of autophagy reduces the proportion of apoptosis Autophagy induced by cytoplasmic Ca^2+^ overload through activating AMPK/mTOR pathway	Autophagy enhances the proportion of apoptotic cells	[55]
		Oral squamous cell carcinoma Cal27	Induction of protective autophagy by promoting ROS pathway and apoptosis	Autophagy reduces the proportion of apoptotic cells	[56]
		Hepatocellular carcinoma Hepg2	Upregulation of beclin1 and LC3-II protein expression and induction of protective autophagy The mechanism may be that it suppresses the AKT/mTOR signal pathway	Induction of protective autophagy	[57]
		Ovarian cancer HEY, A2780	The activation of ERK might cause protective autophagy and apoptosis	Autophagy reduces the proportion of apoptotic cells	[58]
		Hepatocellular carcinoma SMMC-7721, Bel-7402	Activates UPR response and ER stress, and upregulates the expression of eIF2 receptor and CHOP Induces protective autophagy and apoptosis by ER stress	Autophagy induction reduces cell apoptosis	[59]
Dihydroflavone	Chrysin	Glioblastoma TMZ-resistant cell GBM8901	Overcomes MDR through reducing the maturation of LC3-II, ATG12-ATG5 conjugate, and the expression of ATG7 and BECLIN1, inhibiting MGMT expression and TMZ-induced autophagy Inhibition of autophagy by mTOR signal independent pathway	Autophagy triggers the occurrence of MDR	[60,61]
Flavonols	Quercetin	Breast cancer MCF-7, MDA-MB-231 Female BALB/c nude mice	Downregulates MMP-2, MMP-9, and VEGF expression Decreases the level of PKM2, GLUT1, and LDHA and blocks cell glycolysis by inhibiting glucose uptake and the production of lactic acid Suppresses tumor growth, metastasis, and glycolysis through AKT/mTOR-mediated autophagy	Induction of autophagy to inhibit cell migration and invasion	[62]
		Hepatocellular carcinoma SMMC7721, HepG2 Male BALB/c nude mice	Inhibits AKT/mTOR pathway and activates MAPK pathways to induce autophagy In vivo and vitro, induction of apoptotic cells in part via stimulating autophagy	Induction of autophagy to stimulate apoptotic cell death	[63]
		Hepatocellular carcinoma HepG2 (resveratrol + quercetin)	In 8 h, quercetin induces XBP-1 downregulation and CHOP overexpression to abrogate resveratrol-induced ER stress and pro-apoptotic effects In 24 h, resveratrol silences autophagic activation mediated by quercetin, and induces more apoptotic signaling of non-ER stress AMPK phosphorylation, HO-1 downregulation, LMP, and Zn^2+^ dynamics may be the main mechanisms	Induction of protective autophagy	[64]
		Glioblastoma multiforme T98G (quercetin + temozolomide) Anaplastic astrocytoma MOGGCCM (quercetin + temozolomide)	Inhibition of HSP expression results in severe apoptosis and no obvious signs of autophagy, which decreases mitochondrial membrane potential, and increases level of Cyto-c in the cytoplasm and activation of caspase 3 and caspase 9 Activates ER stress, increases level of caspase 12 expression, and changes the shape of nuclei In MOGGCCM cells, autophagic cell death was decreased with the increasing concentrations of quercetin, accompanied by a high level of necrosis and apoptosis	High doses of drugs reduce autophagic cell death and increase apoptotic death	[65,66]
		Glioblastoma U251, U87 Male Sprague Dawley rats	T-AUCB induces overexpression of Atg7 and regulates autophagy-related gene expression Hsp27 inhibitor quercetin suppresses Atg7 expression and strengthens t-AUCB-induced cell death by autophagy blockage	Inhibition of protective autophagy	[67]
		Glioblastoma multiforme T98G (quercetin + sorafenib) Anaplastic astrocytoma MOGGCCM (quercetin + sorafenib)	In MOGGCCM cells, sorafenib mainly initiated apoptosis, and incubating with quercetin potentiated the pro-apoptotic properties of sorafenib In T98G cells, sorafenib mainly initiated autophagy, resulting in an increased number of autophagic cells with quercetin Inhibition of HSP expression increased the induction of apoptosis, but not autophagy	-	[68]
		Hepatocellular carcinoma LM3 Nude mice tumor model	Regulates expression of N-cadherin, E-cadherin, vimentin, and MMP9 Promotes autophagy by inhibiting JAK2/STAT3 signaling pathway and disturbs migration and invasion of tumor cells	-	[69]
		Ovarian cancer CaOV3, P#1 Female nude athymic NOD/SCID mice	Induces protective autophagy and apoptosis through ER stress via the p-STAT3/Bcl-2 axis ER stress is not the only pathway to induce apoptosis in cancer cells	Induction of protective autophagy	[70]
		Primary effusion lymphoma BC3, BCBL1, BC1	Reduces the expression of pro-survival molecules downstream of PI3K/AKT/mTOR, Wnt/β-catenin, STAT3 pathways, and viral proteins involved in KSHV-mediated tumorigenesis Downregulates c-FLIP, cyclin D1, c-Myc, and the release of IL-6 and IL-10 Induces apoptosis and protective autophagy, and increases the cytotoxity of bortezomib	Induction of pro-survival autophagy	[71]
		Burkitt’s lymphoma Akata, 2A8, Ramos	Induces autophagy by inhibiting PI3K/AKT/mTOR pathway and partially degraded mutant c-Myc	Induction of autophagic cell death	[72]
		Gastric cancer AGS, MKN28 Female BALB/c nude mice	Causes autophagy via blocking phosphorylation of Akt, mTOR, and downstream of p70 S6K and 4E-BP1, accumulating HIF-1α HIF-1α enhances BNIP3/BNIP3L to disrupt the interaction of Beclin 1 and Bcl-2/Bcl-xL Induces protective autophagy and apoptosis	Induction of protective autophagy	[73]
		Glioblastoma U373MG	Activates JNK signal, increases the expression and translocation of p53 to the mitochondria, and causes the release of Cyto-c into the cytoplasm Induces protective autophagy and apoptosis	Induction of protective autophagy	[74]
		Pancreatic cancer MIA PACA-2 GEM-resistant MIA PACA-2 ^GEMR^	Knocking down RAGE expression increases apoptosis, autophagy, and GEM-induced cytotoxicity by suppressing the PI3K/AKT/mTOR axis A decrease of NF-κB production may downregulate MDR1 expression	Induction of autophagy to overcome MDR	[75]
		Human T cell acute lymphoblastic leukemia J/Neo, J/BCL-XL	Induces autophagy resulting from attenuating the AKT-mTOR pathway and BCL-XL-sensitive mitochondrial apoptosis	Induction of protective autophagy	[76]
	Galangin	Hepatocellular carcinoma Hepg2	Induces autophagy through the activation of p53 signal pathway	-	[77]
		Laryngeal carcinoma TU212, HEP-2 SPF male BALB/c nude mice	Modulates apoptosis through caspase-3, caspase-9, and PARP cleavage activation and bcl-2 downregulation Suppresses mTOR activation and upregulates TSC1 expression Regulates apoptosis and autophagy by p38 and AKT/NF-κB/mTOR pathways	-	[78]
Flavanols	EGCG	Colorectal cancer HCT-116	Enhances radiation sensitivity through Nrf2 activation and autophagy	-	[79]
		Primary effusion lymphoma BCBL-1, BC-1	Induces apoptosis and autophagy through ROS generation	-	[80]
		Non-small cell lung cancer A549 (gefitinib-resistant cell)/ BALB/C male nude mice	Inhibits autophagy induced by gefitinib and promotes cell death Overcomes MDR through targeting ERK pathway	Inhibition of autophagy to overcome MDR	[81]
Chalcone	Xanthoangelol	Hepatocellular carcinoma Hep3B, HuH7 Male athymic BALB/c nu/nu SPF mice	Increases the expression of LC3-II/LC3-I, Beclin-1, and E-cadherin while decreases p62, N-cadherin, and Vimentin Autophagy induction via the regulation of AMPK/mTOR signal pathway contributes to its anti-metastatic capacity	Suppression of HCC cell metastasis by inducing autophagic cell death	[82]
	Isoxanthohumol	Prostate cancer PC3, DU145	Induces autophagic cell death and cell death is not rescued by caspase inhibitor zVAD-fmk	Induction of autophagic cell death	[83]
		Malignant melanoma B16-F10 Lung metastatic model (female syngeneic C57BL/6 mice)	Induces autophagy and caspase-dependent apoptosis Downregulates integrin α-6, FAK, vinculin, Rho kinase, α-smooth muscle actin, and β-Actin expression, interrupting the integrin signaling pathway In vivo, decreases HMGB1 expression, enhances expression of S100 protein, and inhibits lung metastasis	-	[84]
		Malignant melanoma B16, A375 Syngeneic C57BL/6 mice	Induces protective autophagy and apoptosis Induces A375 cells to differentiate and lose their pluripotency by inhibiting the expression of Notch1, β-catenin, and Oct-3/4 and targeting members of the key signals PI3K/Akt and MEK-ERK pathways	Induction of protective autophagy	[85]
	Flavokawain B	Glioblastoma multiforme U251, U87, T98, P3 Male athymic mice	Induces senescence by ER stress-dependent autophagy Regulation of autophagy though ATF-4/DDIT3/TRIB3/mTOR/RPS6KB1 signaling pathway Inhibition of autophagy caused the switch from senescence to apoptosis.	Induction of autophagy to promote senescence	[86]
	Licochalcone A	Breast cancer MCF-7	Increases the expression of LC3-II/LC3-I and caspase-3 activity and decreases Bcl-2 expression Promotes autophagy and apoptosis through suppressing PI3K/Akt/mTOR activation	-	[87]
	Isoliquiritigenin	Human uterine sarcoma Drug-resistant cell MES-SA/Dx5, MES-SA/Dx5-R (doxorubicin-resistant cell)	Inhibition of autophagy by mediating mTOR pathway Enhances the chemosensitivity of multidrug-resistant cells to doxorubicin	Inhibition of autophagy to overcome MDR	[88]
		Breast cancer Epirubicin-resistant cell MCF-7/ADR Female NOD/SCID mice	Induction of autophagy and G2/M checkpoint block, and downregulation of ABCG2 expression, but no induction of apoptosis Induces autophagic cell death through inhibition of miR-25, and upregulation of ULK1 expression Synergistic effect with epirubicin induces autophagy in vivo	Induction of autophagic cell death to overcome MDR	[89]
		Gastric cancer MKN28	Upregulates the ratio of LC3-II/LC3-I and Beclin 1 expression, and downregulates p62 expression Inhibits the proliferation, migration, and invasion of cancer cells with PI3K/AKT/mTOR-mediated apoptosis and autophagy	Suppression of cell metastasis by inducing autophagy	[90]
	Hydroxysafflor yellow A	Hepatocellular carcinoma Hepg2	Induces autophagy through upregulation of Beclin-1 expression, and inhibition of ERK phosphorylation	Induction of autophagy inhibits cell proliferation	[91]
Flavonolignans	Silibinin	Fibrosarcoma HT1080	Induces autophagic cell death and apoptotic death via ROS pathway Induces p53-mediated autophagic death depending on the activation of ROS-p38 and JNK MAPK pathways	Induction of autophagic cell death	[92,93]
		Breast cancer MCF-7	Increases Atg12-Atg5 formation, Beclin-1 and BNIP3 expression, and ROS levels, and decreases the Bcl-2, ΔΨm, and ATP levels Induces autophagic cell death through ROS-dependent mitochondrial dysfunction and ATP depletion involving BNIP3	Induction of autophagic cell death	[94]
		Cervix carcinoma HeLa	Induces ROS/RNS-mediated autophagy NAC, L-NAME, and GSH attenuate the cytotoxicity by decreasing ROS levels and inhibiting both autophagy and apoptosis	Induction of autophagic cell death	[95]
		Renal cell carcinoma ACHN, 786-O	Induces autophagy through activation of AMPK/mTOR pathway, and downregulation of Wnt/β-catenin pathway Autophagy induction contributes to anti-metastatic effects via regulation of E-cadherin, N-cadherin, and vimentin in a concentration- and time-dependent manner	Autophagy induction inhibits cancer cell metastasis	[96]
		Prostate carcinoma DU14 (silibinin + arsenic)	Induces G1 or G2/M phase arrest, and respectively decreases the protein levels of CDK2, CDK 4, CDK 6, cyclin D1, cyclin D3, and cyclin E and increases p21 and p27. Scavenges arsenic-induced ROS, enhancing autophagy signal Represses the motility and invasive potential, and decreases the expression of MMP-2 and vimentin, without any considerable effect on E-cadherin	-	[97]
		Salivary gland adenoid cystic carcinoma GDC066 Lung metastasis model (BALB/c nu/nu mice)	Increases the autophagic bodies and LC3-II levels Inhibits the proliferation and lung metastasis of ACC cells through autophagy induction in a dose- and time-dependent manner	Autophagy induction inhibits cancer cell proliferation	[98]

## Abbreviations

3-MA	3-methyladenine
8-PN	8-prenylnaringenin
ABC	ATP-binding cassette
ACC	Adenoid cystic carcinoma
ACD	Autophagic cell death
AKT	Protein kinase B
AML	Acute myeloid leukemia
AMPK	AMP-activated protein kinase
ATGs	Autophagy-related genes
BL	Burkitt’s lymphoma
BNIP3	Bcl-2 adenovirus E1B 19-kDa-interacting protein 3
CC	Cervical cancer
Cdc25C	Cell division cycle 25c
CDK	Cyclin-dependent kinase
CHOP	C/EBP homologous protein
CKI	Cyclin-dependent kinase inhibitor
CQ	chloroquine
CRC	Colorectal cancer
DAPK	Death-associated protein kinase
DDIT3	DNA damage inducible transcript 3
DRAM	Damage-regulated autophagy modulator
DRP-1	DAPK-related protein kinase
DRR	DNA damage response
ECM	Extracellular matrix
EGCG	Epigallocatechin-3-Gallate
EGFR	Epidermal growth factor receptor
EMT	Epithelial-mesenchymal transformation
ER	Endoplasmic reticulum
FAK	Focal adhesion kinase
FIP200	FAK interacting protein 200kD
FLIP	Fas-associated death domain-like IL-1β-converting enzyme inhibitory protein
FoxO	Forkhead box class O
GC	Gastric cancer
GLUT1	Glucose transporter1
HCC	Hepatocellular carcinoma
HIF-1α	Hypoxia-induced factor 1α
HMGB1	High-mobility group box 1 protein
HO-1	Palatino Linotype
HSPs	Heat shock protein
HSYA	Hydroxysafflor yellow A
IGF-1	Insulin-like growth factor-1
ILK	Integrin-linked kinase
ISL	Isoliquiritigenin
IXN	Isoxanthohumol
Keap-1	Kelch-like ECH-associated protein 1
LA	Licochalcone A
LDHA	Lactate dehydrogenase A
LMP:	Lysosomal membrane permeabilization
lncRNA	Long non-coding RNA
LPS	Lipopolysaccharide
MAPK	Mitogen-activated protein kinase
MDR	Multidrug resistance
MGMT	O6-methylguanine-DNA-methyltransferase
miRNAs	MicroRNAs
MMPs	Matrix metalloproteinases
mTOR	Mammalian target of rapamycin
NAC	N-acetylcysteine
NB	Neuroblastoma
NF-κB	Nuclear factor kappa B
Nrf2	Nuclear factor erythroid 2
NSLC	Non-small cell lung cancer
p-AKT	Phosphorylated Akt
PCGEM1	Prostate cancer gene expression marker 1
PE	Phosphatidylethanolamine
PEL	Primary effusion lymphoma
PI3K	Phosphoinositide 3-kinase
p-IκB	Phosphorylation of inhibitor of kappa B
PKM2	Glycolysis-related proteins Pyruvate kinase M2
PLKs	Polo-like kinases
p-mTOR	Phosphorylated mTOR
PXN	Paxillin
RAGE	Receptor for advanced glycation end products
RCC	Renal cell carcinoma
RCE	*Rhus coriaria* ethanolic extract
RNS	Reactive nitrogen species
ROS	Reactive oxygen species
RPS6KB1	Ribosomal protein S6 kinase B1
SASP	Senescence-associated secretory phenotype
sEHi	Soluble epoxide hydrolase inhibitor
SIL	Silibinin
siRNA	Small interfering RNA
SIRT1	Silent information regulator 2 homolog 1
t-AUCB	trans-4[-4-(3-adamantan-1-yl-ureido)-cyclohexyloxy]-benzoic acid
TCA	Tricarboxylic acid
TGF	Transforming growth factor
TICs	Tumor-initiating stem cell-like cells
TMZ	Temozolomide
TNF	Tumor necrosis factor
TRAIL	TNF-related apoptosis-inducing ligand
TRIB3	Tribbles pseudokinase 3
UBL	Ubiquitin-like protein
ULK1/2	Unc-51-like kinase
UVRAG	Ultra-violet radiation resistance-associated gene protein
XAG	Xanthoangelol
XBP-1	X-box binding protein 1
XN	Xanthohumol
zVAD-fmk	Benzyloxycarbonyl-Val-Ala-Asp-fluoromethyl ketone
